# Study on carbon emission reduction effect of institutional openness in China

**DOI:** 10.1038/s41598-023-27442-5

**Published:** 2023-01-05

**Authors:** Jinguang Guo, Hongli Wang

**Affiliations:** School of Public Administration, Dongbei University of Finance and Economics, Dalian, 116000 Liaoning China

**Keywords:** Climate sciences, Environmental sciences

## Abstract

As the main means to dovetail the domestic system with international rules, institutional openness is the key to deepening participation in the global economic governance system, breaking through energy and carbon emission constraints, and achieving green and sustainable economic development. Taking 284 prefecture-level cities in China from 2006 to 2019 as the research sample, this paper uses the establishment of Pilot Free Trade Zones as a quasi-natural experiment to systematically identify and test the actual impact of institutional openness on urban carbon emissions in China through the asymptotic difference in difference method, instrumental variables method, spatial econometric model, and mediating effects model. Meanwhile, technological progress is used as the entry point to analyze the intrinsic mechanism of action by adopting digital transformation oriented to efficiency improvement and green innovation capability oriented to R&D innovation as the differentiated perspective. It is found that institutional openness significantly suppresses urban CO_2_ emissions, and there is a certain heterogeneity and spatial spillover effect of this effect. Further study finds that institutional openness achieves carbon emission reduction through technological progress. The study aims to find new institutional innovation and development paths for low carbon development.

## Introduction

Since the industrial revolution, China's urbanization and industrialization have been advancing rapidly. Relying on the low-cost competitive advantages brought by factor dividend and policy tilt, China has quickly embedded itself into the division system of the global value chain, and has achieved many miracles of quantity growth. However, the strategy of giving priority to the development of heavy industry in the past and the relatively loose environmental regulation intensity have also made China's energy consumption and CO_2_ emissions continue to rise, and the ecological environment is close to the red line. In the face of increasingly severe energy and environmental constraints, the 19th National Congress of the Communist Party of China put forward the great development goal of promoting ecological civilization, promoting green and sustainable development, and achieving a beautiful China; In 2020, China made a commitment to the world and announced the dual carbon goal of “Carbon Peak” and “Carbon Neutral”; In the same year, the Fifth Plenary Session of the 19th CPC Central Committee further emphasized the development concept of “Lucid waters and lush mountains are invaluable assets”, and the construction of ecological civilization and green low-carbon development were promoted to an unprecedented height. Accordingly, changing the extensive development model of high-speed growth and exploring the connotative development model of environmental protection and high-quality economic development are the strategic needs of China's sustainable development in the new era^1^. Industrial structure, energy efficiency, urbanization process, economic agglomeration, technological progress, environmental regulation, etc. have also become effective perspectives and ways for scholars and the industry to explore low-carbon emission reduction^2–4^.

However, in the face of the current new development pattern of “domestic circulation as the mainstay and domestic and international circulation promoting each other”, are there new perspectives and explorations that can better respond to the "Carbon Peaking" and "Carbon Neutral" targets scientifically? It is not difficult to find that the existing perspectives and approaches still lack focus on the macroeconomic pattern and background, or at least the response to the macro dimension is indirect. This is not only inconsistent with the strategic requirements of high-quality economic development and ecological civilization construction, but may also cause China to miss an important window of opportunity for low-carbon transition. Therefore, how to directly explore carbon reduction mechanisms and pathways at the macro level will undoubtedly be a major theoretical and practical problem facing the development of low-carbon transition in China at present and in the future. In the macro context, as an important direction to promote the political and economic macro system structure in the dimension of openness up to the outside world, institutional openness not only promotes the deep integration of domestic reform and opening up to the outside world at the institutional level in the new era, but also injects new momentum for China to enhance its international competitiveness. So, can the macro pattern of institutional openness become an important breakthrough and lever for China's low-carbon economic transformation, so as to reverse the traditional development model of “emphasizing economy over environment”? In the face of the new multi-polar international pattern and the serious impact of the new pneumonia epidemic, how to position and develop system openness to unblock the virtuous cycle of the national economy, better form a dual domestic and international circulation pattern, and thus achieve a “win–win” for the two strategies of global system supply and energy conservation and emission reduction?

To address the above questions, this paper takes 284 cities across China from 2006 to 2019 as a research sample to examine the role of institutional openness in carbon emission reduction and its intrinsic influence mechanism. Firstly, based on the continuous and stable nighttime lighting data of time series, we try to scientifically and accurately estimate urban CO_2_ emissions and analyze their evolutionary characteristics. Secondly, based on the development history of institutional openness, we use quasi-natural experimental research methods such as progressive difference in difference (DID) method, instrumental variables method, propensity score matching method and spatial progressive DID method to systematically identify the impact of institutional openness on urban carbon emissions and conduct scientific and rigorous robustness tests. Then, we analyze the mediating effect of technological progress in the impact of institutional openness on carbon emission reduction from multiple perspectives. Finally, from the perspective of “top-level design”, we suggest countermeasures for the green development of institutional cities and even the whole country.

Based on this, this study embeds low-carbon development in institutional space and explores new mechanisms and new ways of carbon emission reduction in the context of institutional openness at both theoretical and empirical levels. The possible contributions of the article are: first, in terms of research perspective, it is the first time to construct a research framework of institutional openness and low-carbon transition, which broadens the depth and breadth of institutional openness research and provides a direct answer to the key question of whether institutional openness can truly serve the high-quality economic development in the new era. Second, in terms of research methodology, based on the traditional DID model, it further combines it with the spatial econometric model in an attempt to improve the general analytical paradigm of spatial DID model and make up for the shortcomings of the traditional DID model estimation framework. Finally, in terms of mechanism analysis, the role of technological progress as an engine for low-carbon transformation is taken as an entry point to analyze the mediating effect of technological progress in the impact of institutional open carbon emission reduction from two perspectives, namely, efficiency enhancement-oriented digital transformation and R&D innovation-oriented green innovation capability, respectively, to seek new development paths of institutional innovation and technological reform for economic low-carbon transformation.

The authors divided the study into seven different parts. The second part finds the gaps in the existing literature based on the literature review. The third part presents the relevant research hypotheses based on the mechanism analysis. The fourth part systematically presents the study design, variable measurement and selection and relevant data description. The fifth part presents the empirical tests, analysis of results and a series of robustness tests. The sixth part discusses the research results. Finally, research conclusions and policy recommendations are presented.

## Literature review

### Institutional openness

#### Background

The 2018 Central Economic Work Conference clearly put forward the concept of “institutional openness”, pointing out that institutional openness is the cross-border integration of rules and standards. The 14th Five-Year Plan of 2021 expands the definition of institutional openness from the level of rules and circulation to four levels of rules, regulations, management and standards, i.e., international and interregional coordination and integration of trade rules and regulations, investment rules and regulations, and management and standards in production to promote openness.

In 1978, after the Third Plenary Session of the Eleventh Central Committee made the major decision to reform internally and open up to the outside world at the same time, Guangdong Province and Fujian Province were taken as model zones to implement “special policies and flexible measures” as a guideline for foreign economic activities, and Shenzhen, Zhuhai, Xiamen, Shantou and Hainan were successively established. In 1984, 14 coastal open cities were established, and some counties under the jurisdiction of the coastal open cities were established as coastal economic open zones, which were the initial exploration and partial experiments to promote institutional openness, and also laid the foundation for China's application for resumption of GATT contracting party status and accession to the World Trade Organization (WTO). The WTO has actively dovetailed with the international economic and trade rules system, deeply participated in the division of labor in the global value chain, and the institutional openness, began to enter the stage of active participation and integration. Meanwhile, it has significantly reduced trade tariffs, eliminated non-tariff barriers such as import quotas, import licenses and specific tenders, further coordinated the two major international and domestic markets, and continuously deepened the openness up of industrial areas to provide guarantees for the liberalization of trade in goods and investment. In 2013, with the introduction of the “One Belt, One Road” initiative and the establishment of PFTZs, China has formed an all-round, multi-level and multi-disciplinary pattern of openness up to the outside world. China has begun to explore and gradually achieve deeper integration with international economic and trade systems and management standards, and further promote the construction of an open economic system.

#### Pilot free trade zones (PFTZs)

**Figure 1:**
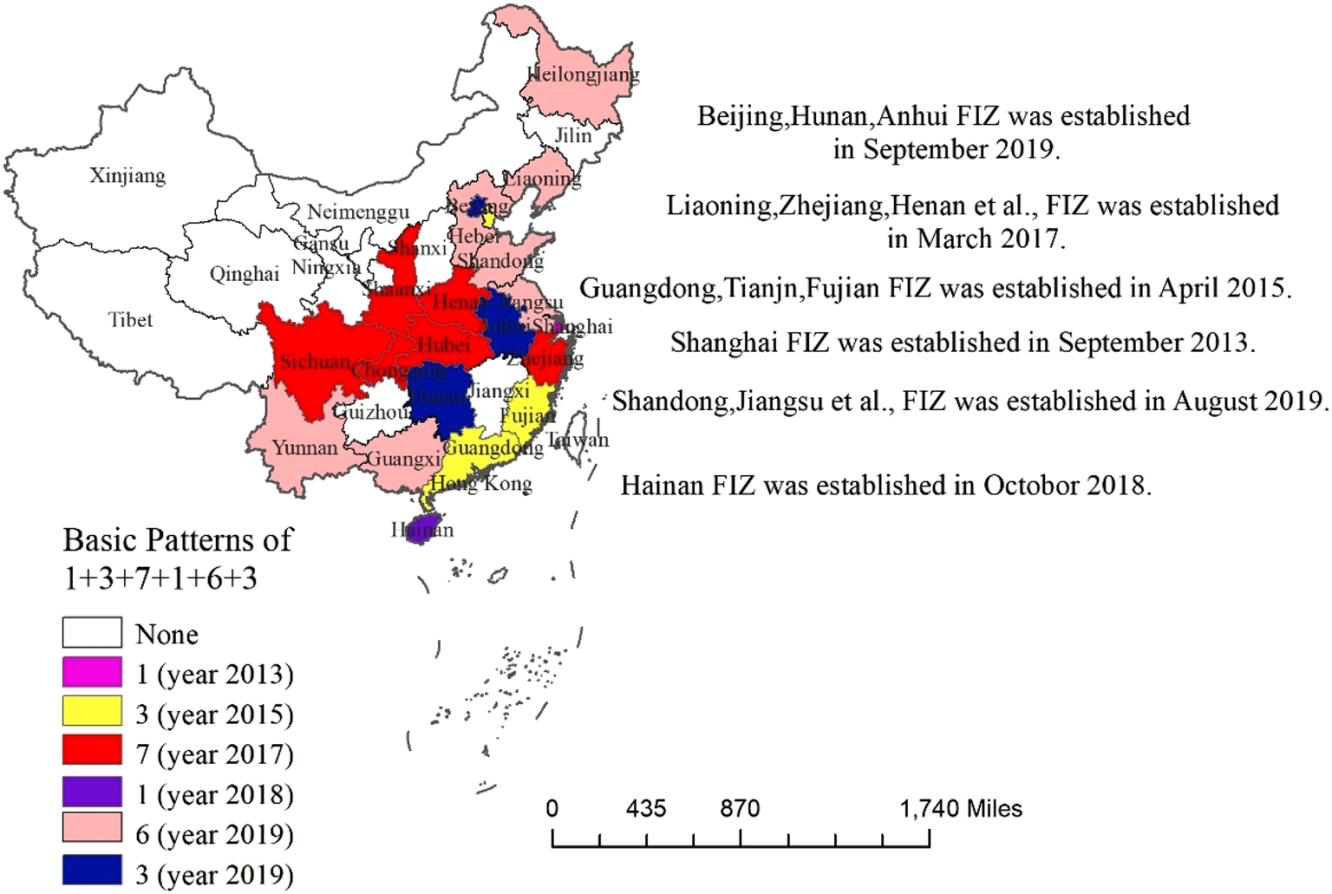
The time and spatial distribution of the establishment of PFTZs in different regions of China. *Source* ArcMap 10.7.010450 https://desktop.arcgis.com/zh-cn/arcmap/.

Promoting the implementation of institutional openness, is an important platform for China to actively participate in the formulation of international economic and trade rules and to fight for institutional power in global economic governance, and it is also the key to promote the green and high-quality transformation of the economy, and thus to realize the Chinese dream of the great rejuvenation of the Chinese nation. Among them, the establishment of the Pilot Free Trade Zone (PFTZs) is an important symbol of entering the stage of comprehensive exploration and innovation leadership of institutional openness, which has more preferential trade arrangements in terms of trade and investment, and sets aside specific areas outside the customs borders of sovereign countries or regions to grant free access to foreign goods without tariffs. The setting of the PFTZs has given a strong impetus to the investment management system, improved trade facilitation, financial openness innovation and transformation of government functions. As the frontier of institutional openness, PFTZs bear the heavy responsibility of early and pilot implementation of international economic rules^5^. 2021, the twentieth meeting of the Comprehensive Deepening Reform Commission further emphasized that “we should accelerate the institutional openness such as rules and standards, improve the layout, and build a higher level of A new system of open economy”. By the end of 2021, China has set up PFTZs in 21 provinces (municipalities directly under the central government) in six batches, forming a new pattern of "1 + 3 + 7 + 1 + 6 + 3" (Fig. 1) and gradually developing into the “leader” of the new round of institutional innovation, and gradually developed into the "leader" of the new round of institutional innovation, involving nearly 90 zones and 260 reform and opening-up experiments, forming a coordinated form of opening-up by land and sea, east and west, north and south, and promoting the formation of a new pattern of China's new all-round institutional opening-up.

Academic quantitative research on PFTZs has focused on the exploration of their economic benefits and innovation-driven effects. Krugman argued that the establishment of PFTZs promotes the free flow of production factors, reduces trade costs, liberalizes trade, and thus promotes economic growth^6^. Yao and Whalley also pointed out that trade openness brings interest rate volatility and economic liberalization, which accelerates the process of capital liberalization and enhances economic development^7^. Waugh and Ravikumar argued that the establishment of PFTZs increases the country's exports by attracting foreign investment and promotes the country's economic development^8^. Seyoum and Ramirez, from an innovation perspective, pointed out that a good institutional environment in PFTZs strengthens the knowledge spillover effects and provides a basis for clustering innovation capabilities^9^, and Isabel and Marta (2015) reached similar conclusions^10^. Akbari et al. (2019) found that PFTZs can effectively combine domestic production factors with international advanced technologies and have a high level of innovation-driven effects^11^.

### CO_2_ emissions

#### CO_2_ emission measurement method

Quantitative studies on carbon emission-related measurements mainly include total amount, intensity and performance. Among them, scholars have estimated carbon emission levels from different geographical scales and study subjects. At the regional level, Muhammad and Khan explored the carbon emission levels of 34 Asian host countries and 115 source countries during 2001–2012^12^. Li and Wei estimated the carbon emissions of 30 provinces and regions in mainland China (excluding Tibet) based on the carbon emission factors published by the Intergovernmental Panel on Climate Change (IPCC, 2006) and found that there were significant regional differences in provincial CO_2_ emissions in China, and CO_2_ emissions were significantly higher in the northeast than in their regions^13^. Xu et al. estimated total factor carbon emission performance by using data envelopment analysis (DEA) method with Chinese prefecture-level city panel data and carbon emissions as non-expected outputs, which involved data mainly from the China carbon accounting database (CEAD) (https://www.ceads.net/data/county/)^14^. Wang and Guo also empirically analyzed the overall changes, spatial and temporal patterns, and regional variability of CO_2_ emissions in 272 cities in China based on nighttime lighting data from this database^15^. At the sector and industry level, Zhang and Wei measured the carbon emission performance of the transportation sector in China from 2000 to 2012 and found that the carbon emission performance of the transportation sector showed an increasing trend^16^. Wang et al. found higher carbon emissions from the manufacturing sector in the Pearl River Delta cities in China^17^, and Cui et al. also reached similar conclusion^18^. Wu et al. used three industrial land spaces (agricultural, production and living and transportation spaces) in China from 1997 to 2016 to reveal the spatial and temporal distribution of annual emissions from industrial land^19^.

#### CO_2_ emission influencing factors

Research perspectives on the factors influencing carbon emissions are also complex and diverse. Chambers and Nakicenovic identified land use changes driven by socio-economic dynamics (e.g., population growth and urbanization) as a major tributary influence on carbon emissions^20^. Wang et al. did a similar study and found that population urbanization is a key factor affecting regional environmental quality^21^. Shahbaz et al. and Li et al. pointed out that raising energy prices and optimizing energy consumption structure are effective ways to improve the environment^22,23^. Du et al. analyzed the effect of green innovation on carbon emission performance using panel data of 71 economies from 1996 to 2012, and found that the effect of green innovation on carbon emission performance has some income threshold effect^24^. Jiao et al., Cheng et al. and Cui et al. reached similar conclusions that into technological innovation not only contributes to economic growth and resource optimization, but also reduces carbon emissions^25–27^. The level of marketization, industrial structure, fiscal decentralization and financial development have also been shown to be key factors influencing carbon emissions^13,28–30^. In addition, a series of changes triggered by the internet have propelled human society into the digital era, and the role of the digital economy on low carbon emission reduction has been widely noticed^31–33^.

### Institutional openness and CO_2_ emissions

Institutional openness is an important symbol to promote a high level of openness to the outside world, which is a major judgment made by the state based on the changes in the domestic and international political and economic environment as well as the development trend, and is an inherent requirement for China's economy to step into high-quality development. Forslid et al. pointed out that institutional openness significantly reduces carbon emissions through investment effects^34^. Feng et al. trade facilitation brought by institutional openness can promote the extension of upstream and downstream industrial chains, which helps share intermediate products among different enterprises and reduce marginal abatement costs and pollution control costs^35^. Also, the specialized division of labor formed by the continuous improvement of industrial chains will also enhance the efficiency of resource allocation, reduce energy consumption and curb CO_2_ emissions^36^. Moreover, institutional openness provides a platform for international knowledge and technology exchange, which can achieve low-carbon technology absorption, learning, innovation and spillover, thus alleviating regional carbon emission reduction pressure^37^.

### Literature gaps

In summary, we find three gaps in the existing research: first, the existing literature has achieved remarkable results on carbon emission and its influencing factors, but it is mainly based on the influence of traditional factors such as industrial structure, energy structure and innovation capacity, and almost no literature explores it from the perspective of institutional openness; Second, less literature compares and quantitatively studies the development history of institutional openness, and there are great limitations in the methodology. Third, when exploring the environmental effects of institutional openness, the analysis of the influence mechanism is insufficient, especially the analysis of the path of technological progress in the process of institutional openness in low carbon emission reduction, which may be lacking in systematization and comprehensiveness.

In order to fill these gaps, this paper is improved as follows: first, based on the macro context, a theoretical analysis framework of institutional openness and carbon emissions is constructed to explore the carbon emission reduction effect of institutional openness; Second, on the basis of sorting out the development history of institutional openness, an asymptotic difference in difference (DID) method, instrumental variables method, propensity score matching method and spatial asymptotic DID method are adopted as exogenous policy shocks in the PFTZs and other quasi-natural experimental research methods to conduct systematic analysis and a series of robustness tests to obtain more scientific and accurate research results. Third, taking technological progress as the entry point, we analyze the mediating effect of technological progress in the impact of institutional openness on carbon emission reduction from two perspectives: digital transformation oriented to efficiency improvement and green innovation capability oriented to R&D innovation, respectively, in order to explore the impact paths of both.

## Theoretical mechanisms and research hypotheses

### Institutional openness and carbon emissions

Energy saving and institutional openness have gradually become the two main themes in China's high-quality transformation process, so embedding carbon emission reduction into the vision of institutional openness and thus constructing a system of issues between the two will help both green development and institutional advancement. As a milestone process of institutional openness, the Pilot Free Trade Zone (PFTZs) requires trade openness to be both prosperous and green, focusing on the joint enhancement of economic and environmental benefits. Specifically, first, the establishment of PFTZs has broken the inherent trade barriers and formed a network. The reduction or cancellation of tariffs has unblocked the trade barriers caused by geographical transportation distance. The enterprises of network member countries can break through the restrictions of geographical distance, form closer trade exchanges, and promote the extension of the global value chain and industrial division and cooperation. More convenient, more efficient, lower cost trade policy and more concise access negative list policy have significantly increased the scale of foreign trade and investment, thus affecting the regional environmental quality. And cities with larger economic scale have greater efforts to environmental governance^38^. On the one hand, the expansion of economic scale can effectively promote the flow of factors and industrial agglomeration, significantly increase the fiscal revenue of local governments, and enhance the fiscal expenditure on low-carbon governance in the region; On the other hand, the expansion of economic scale can force the government to transform to low-carbon development by improving the living standard of local residents and increasing their demand for environmental quality. Second, the overall plan for the establishment of the PFTZs has made clear provisions on low-carbon environmental protection, which makes the network members more inclined to expand the scale of industries with high actual added value and low pollution intensity when conducting trade exchanges and project cooperation. With the deepening of the upgrading of the industrial structure, enterprises with high energy consumption, high pollution and low added value are gradually eliminated, factors of production gradually flow to sectors with low carbon emissions^39^. In addition, with the concept of sustainable development deeply rooted in the hearts of the people, cities also pay more attention to the use of green and clean energy, improve energy utilization efficiency, accelerate the transformation of energy structure, and achieve the carbon emission reduction goal^40^. Last, the establishment of the PFTZs will help domestic enterprises better integrate into the international market, acquire cutting-edge scientific and technological innovation technologies, and carry out secondary innovation, so as to improve the production capacity and efficiency of domestic enterprises, reduce the waste of resources and energy, and achieve the goal of energy conservation and emission reduction^41^. Based on the above three aspects, this study proposes the H1.

H1. Institutional openness will curb urban carbon emissions.

### Spatial effect and heterogeneity of institutional openness on carbon emissions

Geoeconomics points out that there is correlation between everything, including correlation and heterogeneity. Differences in geographical location, city size, factor endowment, economic development level and other factors highlight the heterogeneity between cities^42^. Considering the particularity of foreign trade, differences in institutional environment, geographical location and talent supply will significantly affect the emission reduction effect of institutional openness. Various regions in China have extensive and close links, especially under the influence of "learning effect", “imitation effect” and “economic correlation effect”, the economic activities and policy implementation between spatially related cities usually show an obvious correlation, which is particularly evident in environmental governance policies^43^. Therefore, when examining the impact of institutional openness on carbon emissions, it is necessary to test its possible spatial relevance. In this way comes the hypothesis H2 and H3.

H2. The impact of institutional openness on carbon emissions is heterogeneous.

H3. Institutional openness has a spatial effect on carbon emissions.

### The mechanism of the impact of institutional openness on carbon emissions

From the perspective of technological progress, how does institutional openness achieve carbon emission reduction? This study attempts to analyze the intermediary effect of technological progress in the impact of institutional openness on urban carbon emissions from the perspectives of efficiency oriented digital transformation and R&D oriented green innovation capability.

In the current digital era, as an equally important factor of production as labor, land and capital, the flow of data has become an important basis for international trade^44^. The new round of scientific and technological revolution has brought a huge impact on the global economic governance order. Digital technology, such as artificial intelligence, cloud computing, big data and blockchain, have been widely used in global trade transactions, changing the shape of the global economy to a large extent. Digital technology has gradually become an important driving force for a new round of globalization. Especially in the context of the COVID-19 epidemic, the deep integration of digital technology and trade development has also become an important way to overcome the global economic downturn and green economic development^45^. The institutional openness of digital empowerment unblocks the cross-border transmission and trading channels of digital trade, reduces the cost of enterprises in search, communication and transportation in foreign trade activities, eliminates the geographic distance restriction at the time of delivery, further promotes the scale expansion of digital trade and transformation, improves the production efficiency and resource utilization, and accelerates resources flow to green production sectors with high efficiency and low emissions, thus boosting the low-carbon development of international trade. Moreover, trade facilitation brought by the opening up of the system provides opportunities for domestic and international technology exchanges and promptes the international and domestic emerged many new forms, new model and new technology, has given rise to a series of contribute to CO_2_ capture and storage, a low-carbon clean utilization of energy technical service. In addition, institutional openness advantageous to introduce and absorb advanced production technology, green finance, insurance and other services, which is directly or indirectly reduce urban carbon emissions, thus pushs our country to realize the excess from reducing carbon intensity and unit energy consumption carbon emissions to reduce overall carbon emissions level.

**Figure 2:**
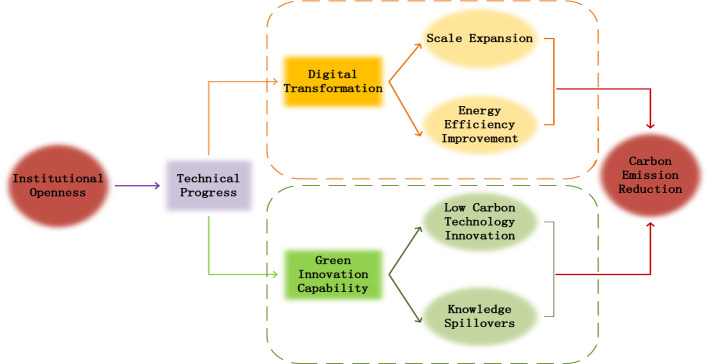
Mechanism analysis.

Institutional openness provides an effective way for China to learn from green production technology and cleaner production experience, fully learn the positive externalities brought by the effect, and provide technical support for its own economic green transformation through the cycle of technology interaction, technology transfer, technology absorption and technology innovation^46^. In addition, the increase of international trade activities will also bring huge economic benefits, provide necessary financial support for green innovation research and development, and also increase the efforts of government departments on pollution prevention and control and governance^47^. In addition, increasingly strict international and domestic environmental regulations will also force enterprises to pay more attention to green R&D and innovation, improve their independent innovation ability, and carry out green and clean open trade activities (Fig. 2). So, we propose the hypothesis H4.

H4. Technological progress has a mediating effect on the impact of institutional openness on urban carbon emissions, and it plays a role through digital transformation and green innovation capability improvement.

## Methodology and data

### Methodology

In order to identify the causal relationship between institutional openness and urban carbon emissions, this study takes the establishment of Pilot Free Trade Zones (PFTZs) as an exogenous policy shock, and uses the difference in differences (DID) method to identify and test. The basic idea of the DID method, which is an important measure to assess the effect of policy implementation, is to consider institutional change and new policy implementation as a “natural experiment” or “quasi-experiment” exogenous to the economic system. The implementation of China's PFTZs may lead to differences in carbon emissions and trade activities in the same pilot city before and after the implementation of the policy; It may also lead to differences in the above two indicators between pilot and non-pilot cities at the same point in time, and the model regression estimation based on these dual differences can effectively control for the effects of other co-temporal policies and the ex-ante differences between pilot and non-pilot cities. The model regression estimation based on this dual difference can effectively control for the impact of other covariate policies and the exante difference between pilot and non-pilot cities, and thus identify the net impact of policy shocks on carbon emissions of cities. Therefore, China's PFTZs policy can be considered as a “quasi-natural experiment”, and its policy effects can be assessed using the DID method. Considering that the traditional DID model is only effective for evaluating the policy effect at a single fixed time point, it is not applicable to evaluating the effect of progressive policies. Based on the research of Callaway and Sant’Anna^48^, a multi-phase DID model was built to compare and analyze the differences of carbon emissions in pilot and non pilot cities. The specific measurement model is as follows:

1 $$ CO_{2it} = \alpha_{0} + \alpha_{1} Policy_{it} + \alpha \sum {X_{it} } + \mu_{i} + \eta_{t} + \varepsilon_{it} $$

where *CO*_*2it*_ is the dependent variable, indicating the carbon emissions of *i* city in t year; *Policy*_*it*_ is the core independent variable, taking the value of 0 before the establishment of the PFTZs and 1 after, and $\alpha_{1}$ represents the estimated coefficient; *X*_*it*_ is the ensemble of control variables; Where $\mu_{i}$ and $\eta_{t}$ denote individual and time-fixed effect, which eliminate the effects of individual heterogeneity and time on the explanatory variables. And $\varepsilon_{it}$ is the random error term.

### Variable selection and data description

#### Independent variable

CO_2_ emissions (CO_2it_). It is confirmed that measuring China's CO_2_ emissions is the basis for achieving energy conservation and emission reduction. However, at this stage, most of the academic measurement of China's CO_2_ emissions is focused on the total carbon emissions calculated based on the carbon emission factors released by the IPCC, and this data only stays at the provincial level, and there is almost no preparation of urban carbon emission inventory. Therefore, in order to accurately measure the level of CO_2_ emissions in prefecture level cities and reduce measurement errors, this study uses particle swarm optimization back propagation (PSO-BP) algorithm to calculate urban CO_2_ emissions based on the night light image remote sensing data set^49^. The nighttime lighting data exclude the influence of fire and other accidental events, as well as the influence of natural factors such as sunlight, moonlight, aurora and clouds, so as to minimize the interference of human factors, with high reliability. Moreover, considering that human production and operation activities will produce a large amount of CO_2_, and the nighttime lighting data covers urban nighttime, residential areas and traffic lights, which can relatively accurately reflect the basic information of human activities, and it is reasonable to use it to estimate. There are three types of night light remote sensing images that are freely available and stable worldwide, and their archiving time is 1992–2003, 2011 to date and 2018 to date respectively. Therefore, in consideration of different sensor parameter settings, working modes and time series, this study combines DMSP/OLS and NPP/VIIRS night light data, reclassifies NPP/VIIRS data on the basis of a series of corrections to DMSP/OLS image data, and finally uses the overlapping area of the two image data in space–time to further linearly fit NPP/VIIRS data, The continuous and stable night light data of time series are obtained, and then applied to the estimation of urban CO_2_ emissions.

#### Core dependent variable

The DID item of the PFTZs (*Policy*_*it*_). If the PFTZs are set up in the city and its observation time is later than the selected year, then *Policy*_*it*_ = *1*, otherwise *Policy*_*it*_ = *0*.

#### Control variables

The important impact of economic development on carbon emissions has been widely recognized by the academic community^50^^.^ As an important indicator to measure the level of urban economic activity, the higher the level of GDP per capita is, the greater the role it plays in promoting the market size, and thus the level of carbon emissions. Therefore, GDP per capita is used as one of the control variables in this study. According to EKC hypothesis, there is an inverted U-shaped relationship between environmental quality and economic growth. Therefore, this study adds the quadratic term of GDP to the control variables.

As one of the important driving forces for economic development, external dependence can absorb green technology through “knowledge spillover effect”, thereby alleviating the pressure of environmental pollution; However, enterprises with high emissions and high pollution may also be introduced in pursuit of economic benefits, which will aggravate regional environmental degradation^51^. This study takes the ratio of foreign direct investment to GDP as one of the control variables affecting carbon emissions.

The industrial structure will also have a certain impact on urban carbon emissions. Compared with agriculture and the service industry, the secondary industry is more dependent on energy. In particular, the industrial industry will produce a large amount of carbon emissions in production, which seriously restricts regional green development^52^. This study uses the ratio of the output value of the secondary industry to GDP to measure the industrial structure.

As the basis of regional development, factor endowments determine the urban industrial structure, economic activities and energy use, thus affecting the level of urban carbon emissions^53^. This study measures the proportion of fixed asset investment in GDP.

Considering that the environment is a kind of public goods, the property rights involved cannot be clearly defined, and the obvious externalities may lead to serious market failure^54^. We describe the government intervention behavior with government financial expenditure, and explores its impact on urban carbon emissions.

Financial development is also closely related to the level of CO_2_ emissions^55^. Financial development provides necessary financial support for the R&D and promotion of green innovative technologies, and alleviates environmental pressure through the effect of technological progress. And the developed financial system is easier to promote the development of green finance and unblock new channels for financial development to support energy conservation and emission reduction; Meanwhile, financial development prefers to go to state-owned enterprises with high stability but weak pollution emission control system, which will increase CO_2_ emissions.

In addition, population is also a key factor affecting urban carbon emissions. On the one hand, the rapid increase in the level of human activities will generate more energy consumption, housing and other resources and living needs, and the increase in production and living costs may produce a "congestion effect", which will aggravate regional carbon emissions^23^. On the other hand, population agglomeration will also reduce carbon emissions and relieve the pressure on the ecological environment through "sharing benefits" and "scale effect"^56^. In this study, the population density level is taken as the proxy variable index of population size.

#### Intermediary variable

This study measures the level of technological progress from two perspectives: digital transformation and green innovation capability. Among them, digital transformation is efficiency enhancing technological progress closely related to the ecological environment, while green innovation capability reflects R&D innovative technological progress.

We take the digital economy development level index as the proxy variable of digital transformation^57^. Specifically, select the number of mobile phone users at the end of the year, post and telecommunications business income, Internet broadband access users, and the number of employees (including information transmission, computer services and software industry), and use the principal component analysis method to process the four indicators to obtain the final digital economy development level index. The measurement of green technology innovation is mainly the number of green patents used. The number of green patents includes the number of applications and the number of authorizations. The number of patent applications, to a certain extent, only reflects the importance of green technology, but does not represent the improvement of the actual technology level. Therefore, according to the international patent classification code for green patents in the List of Green Patents issued by the World Intellectual Property Organization, this study connects the number of green patents in the patent database of the State Intellectual Property Office of China, and searches the number of green patents granted in each city according to the patent authorization date to measure the level of green technology innovation.

#### Data sources

The sample used in this study is 284 prefecture level cities' balanced panel data from 2006 to 2019 to evaluate the impact of PFTZs on urban carbon emissions. Relevant data source: website of National Oceanic and Atmospheric Administration (https://www.ngdc.noaa.gov), China Statistical Yearbook, China Urban Statistical Yearbook and relevant policy documents of the State Council. For some cities with missing data, the smoothing index method and linear fit and sum method are used to fill in the data. To alleviate the interference of inflationary factors on the data, all monetary indicators are deflated to constant prices based on price indices in this paper with 2000 as the base period. Considering that the interference of factors such as dimensionality and heteroscedasticity may affect the accuracy of the model. The data of each variable are described in Table 1.

**Table 1:** Data description.

Index	var		Obs	Mean	Std	Min	Max	Unit
*CO*_*2*_	Carbon emissions	Calculate Value	3976	31.581	30.503	1.294	207.634	10,000 tos
*pgdp*	Economic development level	GDP/ Total Population	3976	33,369.74	24,001.66	2501.65	339,893.6	Yuan / people
*sec*	Industrial structure	Output value of tertiary industry / Output value of secondary industry	3976	0.469	0.111	0.090	0.898	%
*fdi*	Openness	FDI/GDP	3976	0.023	0.034	0.000	0.735	%
*gov*	Government intervention	Government Expenditure	3976	0.240	0.310	0.011	6.125	10,000 yuan
*fae*	Factor endowment	Investment in Fixed Assets / Labour Force	3976	20.774	12.586	1.103	85.691	Yuan / people
*per*	Population	Total urban population	3976	431.801	336.929	4.700	2759.139	10,000 people
*fina*	Financial development	Deposit and Loan /GDP	3976	2.825	3.587	0.001	125.629	%

## Empirical results

### Analysis of typical facts

**Figure 3:**
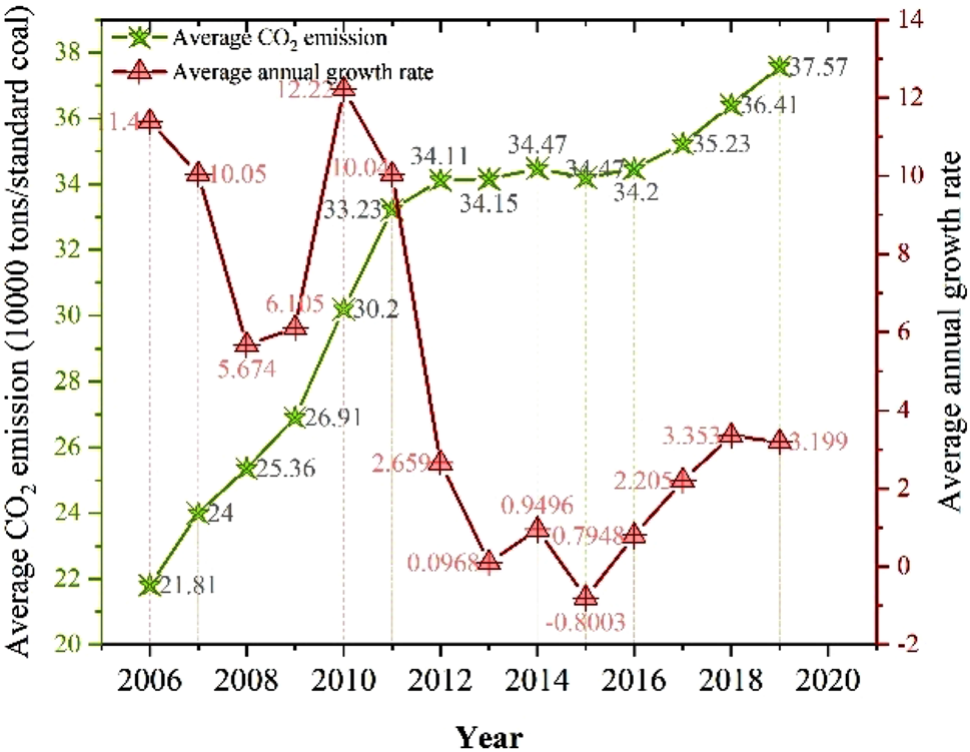
National average CO_2_ emissions and growth rate.

**Figure 4:**
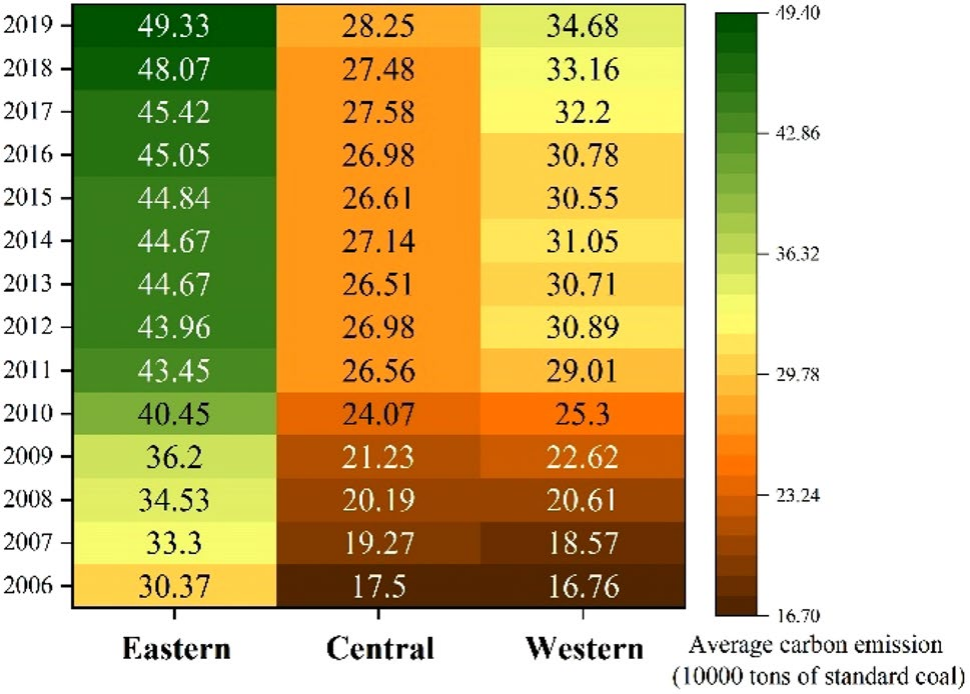
Regional average CO_2_ emissions.

**Figure 5:**
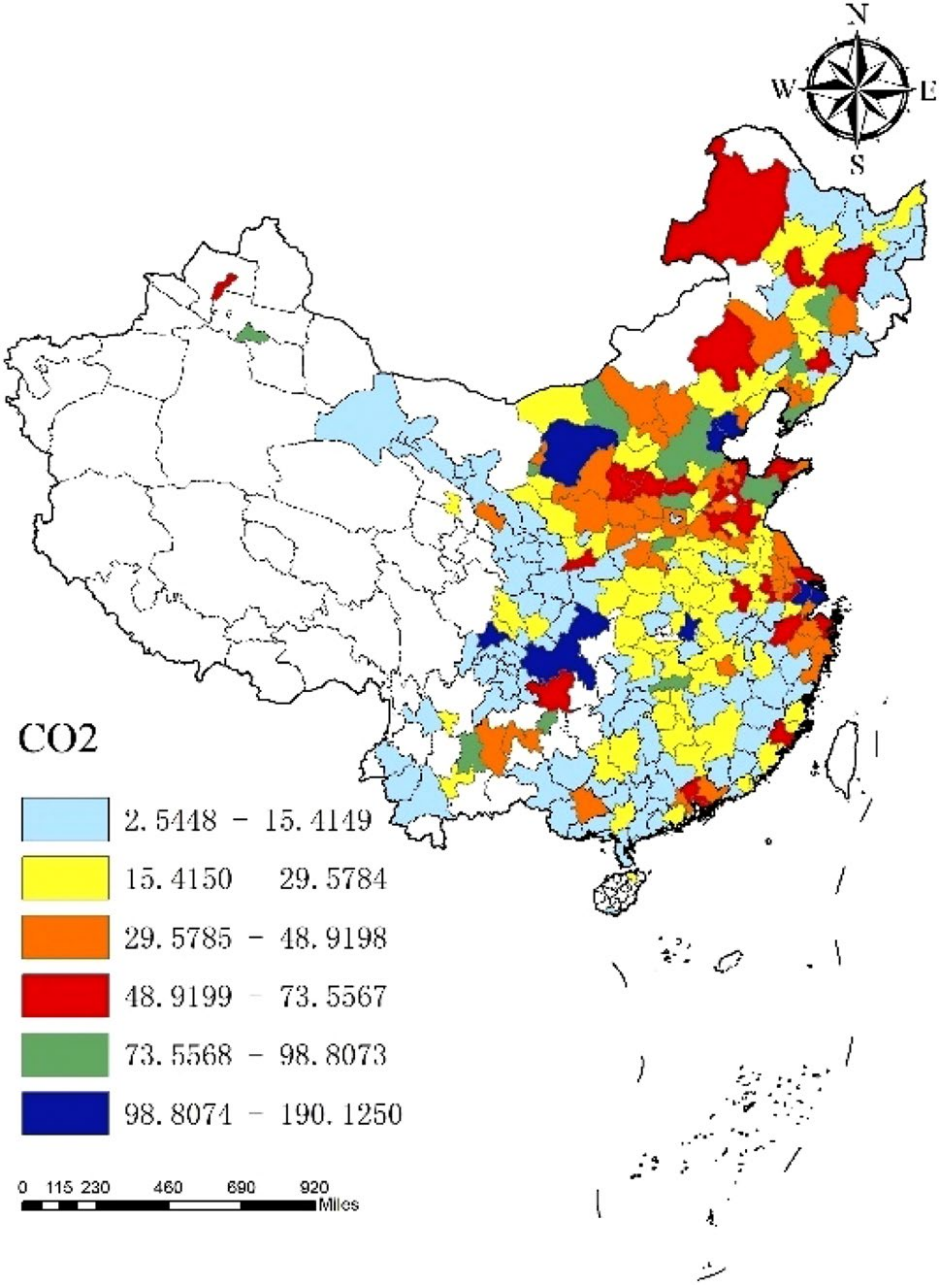
Average CO_2_ emissions. *Source* ArcMap 10.7.010450 https://desktop.arcgis.com/zh-cn/arcmap/.

**Figure 6:**
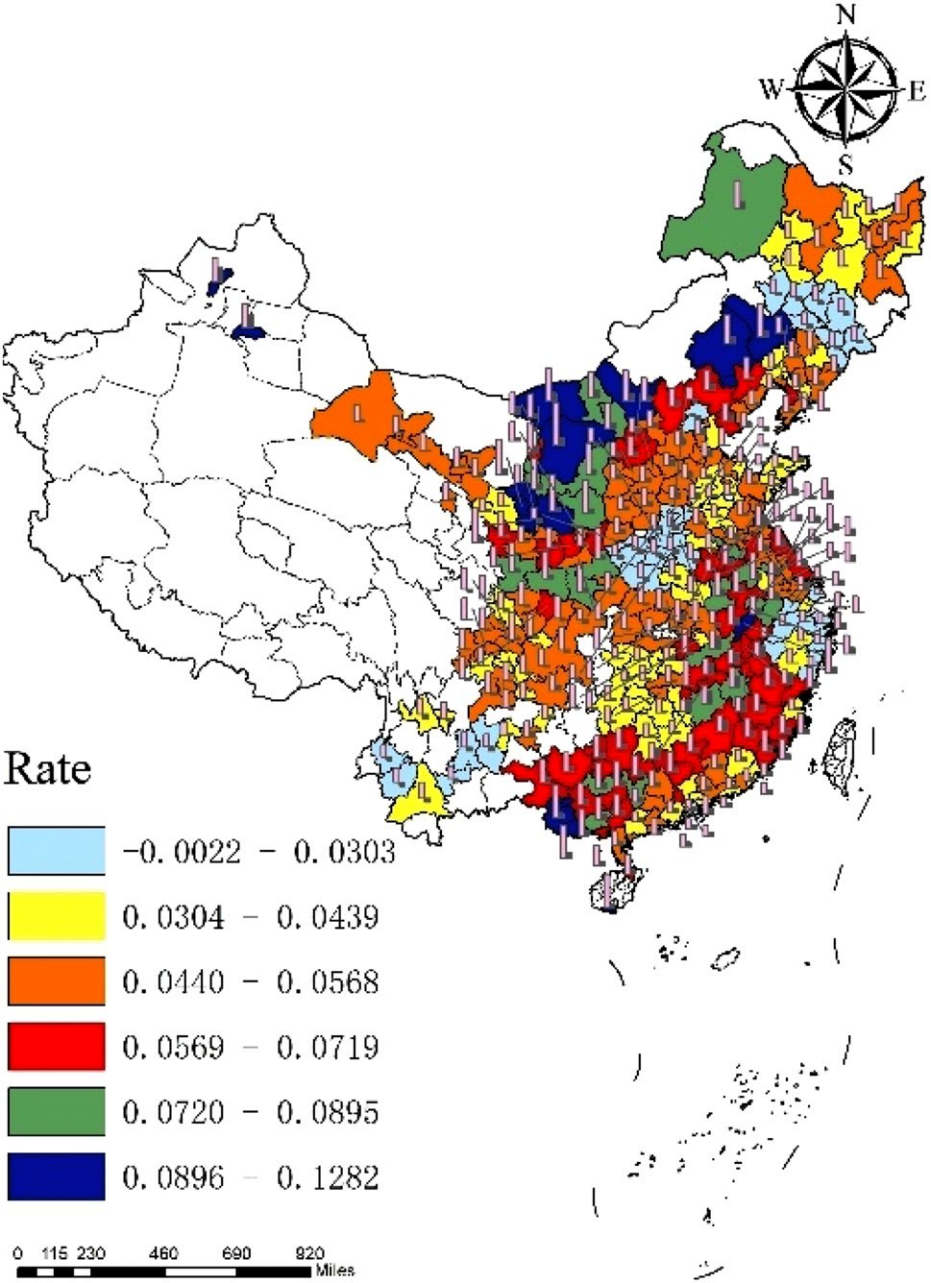
Average growth rate of CO_2_ emissions. *Source* ArcMap 10.7.010450 https://desktop.arcgis.com/zh-cn/arcmap/.

In general, CO_2_ emissions of 284 cities in China fluctuated from 2006 to 2019, with an average annual emission of 307.8 thousand tons and an annual growth rate of 4.85%. From the perspective of cities, the CO_2_ emissions of each city generally show an upward trend, but the fluctuation range is small (Fig. 3). Shanghai ranks first in the country in terms of annual CO_2_ emissions, about 1901.2 thousand tons, while Ya’an, Sichuan Province, has the lowest annual CO_2_ emissions, only 25.4 thousand tons. From the perspective of time, except for the negative growth of CO_2_ emissions in 2015, CO_2_ emissions in other years increase to varying degrees. Among them, the growth rate of CO_2_ emissions in 2006–2010 continued to rise, and reached the peak in 2010 (12.22%). After 2011, the growth rate of CO_2_ emissions in cities across the country began to decline, and it achieves negative growth until 2015. However, after 2015, CO_2_ emissions rebound to a certain extent, but the growth rate is relatively small, which is lower than the average value in the sample period. In particular, from 2018 to 2019, the growth rate of CO_2_ emissions in each city is maintain about 3%. As shown in Figs. 4,5,6, from 2006 to 2019, there is a significant spatial imbalance in CO_2_ emissions of each city. The average annual CO_2_ emissions in the eastern, central and western China are respectively 417,400, 2474 and 276,400 thousand tons. The eastern coastal China has the highest CO_2_ emissions, the western China ranks second, and the central China has the lowest CO_2_ emissions. The economic activities in the eastern China are carried out frequently, and the energy consumption is large. In particular, the large population gathering brings huge consumption demand, further increasing CO_2_ emissions. However, from the perspective of growth rate, the western China has the fastest growth rate of CO_2_ emissions in the sample period, reaching 5.8%, while the eastern and central have a small difference in growth rate, 3.87% and 3.85% respectively.

### Baseline results

In this study, the establishment of Pilot Free Trade Test Zones (PFTZs) is regarded as a policy shock, and the causal relationship between institutional openness and carbon emissions is evaluated using the multi period difference in differences (DID) method. At the same time, the stepwise regression method is used to reduce the interference of control variables on the regression results of core independent variables. The results are shown in Table 2. Without adding control variables, the estimated coefficient of the policy dummy variable Policy_it_ is − 0.072, which is significant at the level of 1%. After adding a series of control variables, the estimated coefficient of Policy_it_ is − 0.053, which is still significant at the 1% statistical level. That is, compared with the cities in the non test zone, the establishment of PFTZs reduces the urban carbon emissions by 5.3%. As an important way to integrate into the world political and economic system, institutional openness plays an important role in global economic governance and environmental governance, which may exert carbon reduction effects through four aspects: policy makers, technology developers, investors and the public. First, institutional openness can increase the supply of local institutional public goods through selective absorption of foreign superior institutions. Especially in the current critical period of low-carbon transition, policy makers can introduce high-quality systems that balance economic and environmental benefits through demonstration effects and institutional migration to promote energy conservation and emission reduction. At the same time, institutional openness leads to further economic expansion, and local governments will receive more fiscal revenue, which in turn will increase government funding to combat environmental pollution in the region. Second, for technology innovators, institutional openness provides an effective way to learn green production technologies and cleaner production experiences, and provides technological support for the green transformation of the economy suitable for oneself through a cycle of technology interaction, technology transfer, technology absorption and technological innovation^58,59^. Third, for investors, institutional openness reduces institutional frictions and coordination costs in production cooperation through the harmonization of standards and regulations in the production process. This more convenient, lower cost, and more efficient trade and investment policy attracts foreign capital. The overall program of institutional openness also clearly puts forward higher environmental requirements, raising the environmental threshold for access to pollution-intensive industries, and the inflow of foreign capital increasingly tends to be of high quality, thus curbing the development of pollution-intensive industries and promoting the green transformation of industrial structure. Fourth, from the public's perspective, the institutional openness makes the concept of green environmental protection more deeply rooted and the economic benefits it brings further enhance the residents' demand for a better environment, which not only makes residents inclined to green consumption, but also pushes the government to increase urban environmental management. H1 is verified.

**Table 2:** Baseline results.

	(1)	(2)	(3)	(4)	(5)	(6)	(7)	(8)	(9)
*Policy*_*it*_	− 0.072***	− 0.072***	− 0.070***	− 0.057***	− 0.057***	− 0.056***	− 0.056***	− 0.054***	− 0.053***
	(− 5.62)	(− 5.70)	(− 5.42)	(− 4.21)	(− 4.35)	− 4.14)	(− 4.14)	(− 3.93)	(− 3.91)
*lnfdi*		− 0.026	− 0.053	− 0.084	− 0.084	− 0.092	− 0.092	− 0.121	− 0.121
		(− 0.33)	(− 0.72)	(− 1.15)	(− 1.17)	(− 1.25)	(− 1.14)	(− 1.49)	(− 1.49)
*lnpgdp*			0.074***	0.645***	0.508**	0.507**	0.507**	0.473**	0.473**
			(4.24)	(3.21)	(2.42)	(2.47)	(2.48)	(2.35)	(2.34)
*lnpgdp*^2^				− 0.029***	− 0.023**	− 0.023**	− 0.023**	− 0.021**	− 0.021**
				(− 2.99)	(− 2.35)	(− 2.39)	(− 2.39)	(− 2.24)	(− 2.24)
*lnsec*					0.083***	0.083***	0.083***	0.086***	0.085***
					(2.84)	(2.82)	(2.81)	(2.94)	(2.92)
*lnfae*						0009	0.009	0.011	0.011
						(0.97)	(0.97)	(1.15)	(1.14)
*lngov*							− 0.001	− 0.016*	− 0.016*
							(− 0.03)	(− 1.66)	(− 1.65)
*lnfina*								0.025*	0.025*
								(1.90)	(1.89)
*lnper*									− 0.006
									(− 0.40)
*N*	3976	3976	3976	3976	3976	3976	3976	3976	3976
*City FE*	Yes	Yes	Yes	Yes	Yes	Yes	Yes	Yes	Yes
*Year FE*	Yes	Yes	Yes	Yes	Yes	Yes	Yes	Yes	Yes
*R*^2^	0.808	0.808	0.812	0.816	0.818	0.818	0.818	0.819	0.819

***p < 0.01; **p < 0.05; *p < 0.1, with t values in parentheses, as in the following tables.

For the control variables, the impact of economic development level (lnpgdp) on carbon emissions is positive, while the regression coefficient of its quadratic term (lnpgdp2) is opposite, and both have passed the significance test with a statistical level of 5%, that is, the impact of economic development on carbon emissions presents an inverted "U-shaped relationship", which first rises and then falls, and this conclusion also conforms to the Environmental Kuznets Hypothesis^60^. At the initial stage of economic development, frequent economic activities will not only improve economic benefits, but also bring a lot of environmental pollution problems such as carbon emissions. However, with the improvement of the economic level, the industrial structure has been optimized and the technical level has been improved. Such high-quality economic activities will promote low-carbon emission reduction^43^. The increase of the proportion of the secondary industry (lnsec), financial development level (lnfina) and resource endowment (lnfae) will increase the regional carbon emission level. Among them, the regression coefficients of lnsec and lnfina passed the significance test of 1% and 10% respectively, and the regression coefficients of lnfae were not significant. This may be because China is still in the early stage of economic transformation, the development of the secondary industry, especially the industrial development, is still unable to be completely decoupled from energy consumption, which still aggravates the total regional carbon emissions. Therefore, the further “green transformation” of the industrial structure is one of the key paths to promote China's low-carbon development. Influenced by performance preference, banks and other financial institutions are more inclined to non environment-friendly enterprises, and technology R&D green enterprises face serious financing constraints. Although the environmental awareness of most enterprises in China has been significantly improved, and the green financial policy has gradually matured from the initial stage of exploration, there are still repeated investments and overcapacity, which strengthen the extensive production mode and hinder the realization of carbon emission reduction goals. The regression coefficients of government intervention (lngov) and population density (lnpop) are both negative, but only the coefficient of lngov is significant at the 10% statistical level. With the deepening of the contradiction between natural resources and social development, the country has attached great importance to the development of ecological environment, and emphasized in the report of the 19th National Congress that the construction of ecological civilization is the millennium plan for the sustainable development of the Chinese nation. Governments at all levels have gradually abandoned the original excessive pursuit of administrative performance, and began to find and apply effective tools that give consideration to the dual functions of economic development and environmental protection, fully releasing the welfare effect of government intervention on energy conservation and emission reduction^61^. The R^2^ of models (1)–(9) is basically maintained around 0.8, indicating that the baseline regression of this paper has a good good goodness of fit and the confidence of the results is high.

### Robustness check

#### Parallel trend test

Satisfying the parallel trend assumption is the first step of policy assessment using the DID method, which requires that the explanatory variables in the experimental and control groups have the same trend before exogenous policy shocks, implying that the trends of carbon emissions in the pilot and non-pilot cities should be parallel before policy implementation. Considering that exogenous policies may be subject to different factors such as the basis and intensity of policy implementation, there is a certain time lag. Therefore, this study draws on the research of Beck and uses the event analysis method to build a dynamic model for hypothesis testing^62^. The details are as follows:

2 $$ CO_{2it} = \beta_{0} { + }\sum\nolimits_{\delta = - 1}^{\delta = - 7} {\beta_{\delta } pre_{it} } + \beta cru_{it} + \sum\nolimits_{\delta = 1}^{\delta = 6} {\beta_{\tau } post_{it} + \beta } \sum {X_{it} } + \mu_{i} + \eta_{t} + \varepsilon_{it} $$

**Figure 7:**
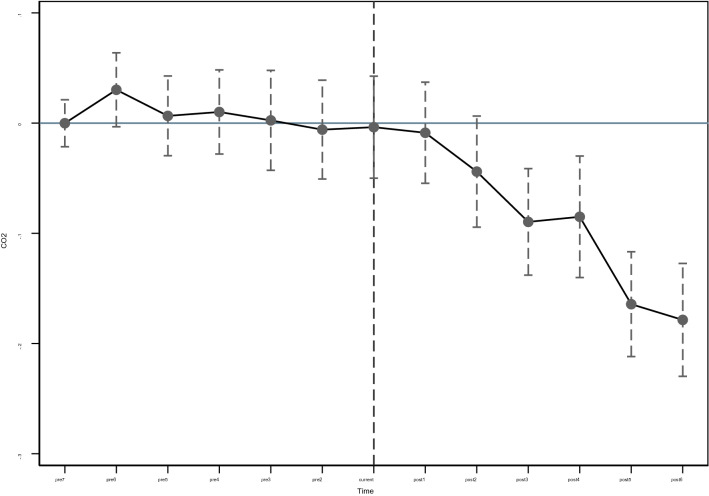
Parallel trend test.

*CO*_*2it*_ is the urban carbon emission level; *pre*_*it*_ and *post*_*it*_ are counterfactual dummy variables. *pre*_*it*_ indicates that before the year of policy implementation, *t* values are − 1, − 2, − 3, − 4, − 5, − 6, and− 7 respectively; Similarly, *post*_*it*_ indicates that after the year of implementation of the pilot policy, *t* values are 1, 2, 3, 4, 5, and 6 respectively; *Cru*_*it*_ indicates the year when the policy is implemented, and the value of t is 0. The settings of other variables are the same as those of the benchmark model. If $\beta_{\delta }$ fails to pass the significance test, it indicates that there is no significant difference in carbon emissions between the control group and the experimental group before and after the implementation of the policy; If $\beta_{\tau }$ passes the significance test, it indicates that after the implementation of the policy, the carbon emissions of the cities in the experimental group and the control group are significantly different. If both conditions are met, the parallel trend test is passed. The settings of other variables (*X*_*it*_, $\mu_{i}$ and $\eta_{t}$) are the same as those of the benchmark model. In order to clearly and intuitively show whether the benchmark model conforms to the parallel trend assumption, this study uses the graphical method to compare the change of urban carbon emissions before and after the establishment of the PFTZs. It can be seen from the test results of parallel trend hypothesis in Fig. 7 that the regression coefficient $\beta_{\delta }$ cannot reject the original hypothesis, that is, before the establishment of the PFTZs, there is no significant difference in carbon emissions between pilot cities and non pilot cities. The regression coefficient $\beta_{\tau }$ rejects the original assumption, that is, after the implementation of the pilot policy, the difference between the carbon emission levels of pilot cities and non pilot cities is growing, which meets the parallel trend assumption. It is reasonable and effective to use the DID method to assess the impact of the policy effect on carbon emissions.

#### Placebo test

**Figure 8:**
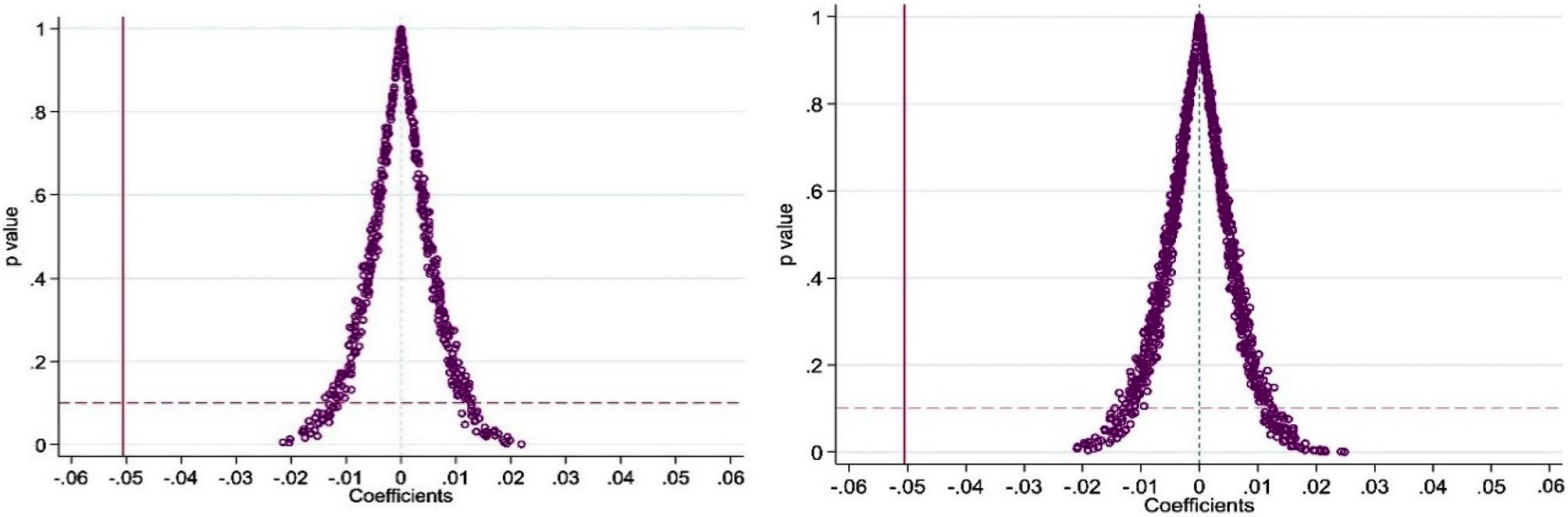
Distribution of *P* values in the placebo test (reference estimates 500 and 1000).

Although this study controls a series of urban characteristic factors that may affect the carbon emission level in the benchmark regression, there may still be some impacts of some unobservable factors that change with time and location, making the estimation result biased. Therefore, the indirect placebo test is used in this study to randomly generate cities in the PFTZs, and 500 and 1000 regression simulations are repeated according to the benchmark model. The specific regression coefficients are:

3 $$ \widehat{{\upbeta }} = \beta + \gamma \times \frac{{{\text{cov}} (Policy_{it} ,\varepsilon_{it} |control)}}{{{\text{var}} (Policy_{it} |control)}} $$

where,$\gamma$ represents the influence of non observation factors. If $\gamma$ = 0, it means that non observational factors do not interfere with the estimation results of the model, that is, $\widehat{{\upbeta }}$ is unbiased. However, it is difficult to obtain the specific value of $\gamma$ through direct test. Therefore, this study uses the variable $\widehat{{\upbeta }}$ randomly produced by the computer to replace *Policy*_*it*_, if $\widehat{{\upbeta }}$ = 0, then $\gamma$ = 0, and the model is accurate; On the contrary, if the model is wrong, the estimation results will be affected by unobservable factors. As shown in the P value distribution diagram (Fig. 8), the estimated values of 500 and 1000 simulated regressions basically follow the normal distribution, which also shows that the regression results in this study have a certain robustness.

#### Non random selection influence test

Considering the non randomness of the setting of the PFTZs, this study introduces a series of interactive items of urban factors and time trends to improve the benchmark model:

4 $$ CO_{2it} = \alpha_{0} + \alpha_{1} Policy_{it} + \alpha \sum {X_{it} } + C_{i} \times Policy_{it} + \mu_{i} + \eta_{t} + \varepsilon_{it} $$

Among them, $C_{i}$ represents four city attribute factors, namely municipality directly under the Central Government, provincial capital city, northern city and sub provincial city. The remaining variables are the same as in Eq. (1).

As shown in the first column of Table 3, the cities in the PFTZs still show a positive impact on carbon emissions after taking into account the urban prerequisites.

**Table 3:** Robustness check.

	Counterfactual test		Non-random factors		Other policy factors		Abnormal value		PSM-DID		
	2 years in advance	4 years in advance					Shrink tail 1%	Shrink tail 2%	Radius matching	Nuclear matching	Nearest neighbor matching
*Policy*_*it*_	0.015	− 0.023	− 0.064***	− 0.048***	− 0.065***	− 0.053***	− 0.058***	− 0.063***	− 0.046***	− 0.042***	− 0.051***
	(1.21)	(− 1.39)	(− 3.59)	(− 3.04)	(− 3.56)	(− 3.63)	(− 4.18)	(− 4.47)	(− 4.40)	(− 4.14)	(− 4.38)
*Municipality directly*			− 0.050								
			(− 1.27)								
*Provincial capital*			0.029								
			(0.95)								
*Sub provincial city*			0.008								
			(0.29)								
*Northern cities*			0.005								
			(0.16)								
*Carbon emission right*				− 0.021							
				(− 0.93)							
*Low carbon pilot cities*					0.024						
					(1.06)						
*Central environmental protection inspector*						0.003					
						(0.27)					
*lnfdi*	0.071	− 0.110	− 0.120	− 0.122	− 0.119	− 0.121	− 0.107	− 0.105	− 0.261	− 0.281*	− 0.620***
	(1.14)	(− 1.33)	(− 1.47)	(− 1.49)	(− 1.46)	(− 1.49)	(− 1.30)	(− 1.32)	(− 1.61)	(− 1.73)	(− 2.69)
*lnpgdp*	0.770***	0.481**	0.477**	0.468**	0.477**	0.473**	0.252	0.163	0.397	0.456*	0.325
	(5.73)	(2.33)	(2.35)	(2.31)	(2.36)	(2.34)	(1.29)	(0.81)	(1.46)	(1.66)	(0.73)
*lnpgdp*^2^	− 0.037***	0.022**	− 0.022**	− 0.021**	− 0.022**	− 0.021**	− 0.011	− 0.006	− 0.018	− 0.021*	− 0.014
	(− 5.88)	(− 2.23)	(− 2.25)	(− 2.21)	(− 2.25)	(− 2.24)	(− 1.14)	(− 0.64)	(− 1.49)	(− 1.67)	(− 0.73)
*lnsec*	0.007	0.086***	0.085***	0.086***	0.084***	0.085***	0.059*	0.045	0.023	0.008	0.013
	(0.41)	(2.94)	(2.91)	(2.94)	(2.87)	(2.92)	(2.03)	(1.36)	(0.70)	(0.25)	(0.25)
*lnfae*	− 0.013**	0.012	0.012	0.011	0.011	0.011	0.014	0.014	0.007	0.006	− 0.004
	(− 2.02)	(1.24)	(1.22)	(1.15)	(1.21)	(1.14)	(1.49)	(1.50)	(0.63)	(0.51)	(− 0.27)
*lngov*	− 0.012*	− 0.018*	− 0.017*	− 0.016	− 0.016*	− 0.016*	− 0.017*	− 0.015	− 0.021**	− 0.022**	− 0.040**
	(− 1.78)	(− 1.76)	(− 1.68)	(− 1.64)	(− 1.67)	(− 1.65)	(− 1.72)	(− 1.49)	(− 2.17)	(− 2.27)	(− 2.21)
*lnfina*	0.012	0.027*	0.025*	0.024*	0.025*	0.025*	0.028**	0.028**	0.032***	0.034***	0.061***
	(1.32)	(1.96)	(1.91)	(1.88)	(1.90)	(1.89)	(2.02)	(1.97)	(2.73)	(2.95)	(− 2.96)
*lnper*	0.023**	− 0.008	− 0.007	− 0.005	− 0.007	− 0.006	− 0.07	− 0.080	0.004	0.006	− 0.039
	(2.53)	(0.52)	(− 0.45)	(− 0.37)	(0.44)	(0.40)	(− 0.44)	(− 0.53)	(0.18)	(0.27)	(− 0.73)
*N*	3976	3976	3976	3976	3976	3976	3976	3976	3976	3976	3976
*R*^2^	0.496	0.818	0.819	0.819	0.819	0.819	0.810	0.800	0.650	0.655	0.652

#### PSM-DID

The establishment of PFTZs in batches may have some problems such as selective errors in the implementation process, which makes the pilot cities and non pilot cities have certain differences in economic development and trade exchanges, thus causing the deviation of regression results. Therefore, this study further adopts the dual difference method of propensity score matching (PSM-DID) to strengthen the comparability between the samples of pilot cities and non pilot cities, and reduce the problem of possible sample selection errors^63,64^. When matching the propensity scores of the samples of pilot cities and non pilot cities, this study uses the idea of prior matching and the mean value of the sample variables before the impact of the policy (before 2013) to match, respectively using three methods: radius matching, kernel matching and nearest neighbor matching. And the selection of covariates is consistent with the control variables. From the results in Table 3, the regression coefficient of the DID item Policy_it_ is significantly negative at the statistical level of 1%, consistent with the benchmark regression results. That is, after matching, the PFTZs still has a significant inhibition on carbon emissions, which also indicates the robustness of the above results.

#### Test of other policy effects

Considering that in the sample period (2006–2019), each city has implemented other policies related to low-carbon governance at different stages, thus reducing the accuracy of the policy evaluation effect in this study. Therefore, this study further controls the impact of representative policies such as carbon emission trading pilot, low carbon pilot and central environmental protection supervision city on urban carbon emissions, and incorporates the dummy variables of the above three policies into the benchmark model for estimation. Among them, the value assigned to selected pilot cities is 1, otherwise it is 0. The results are presented in Table 3. From the results, after controlling the impact of three policies, namely, carbon emission trading pilot, low-carbon pilot and central environmental protection supervision city, the DID sub coefficient (Policy_it_) is still significantly negative at the level of 1%, and institutional openness has a significant inhibition effect on carbon emissions. This paper also considers the effect of outliers, and the above conclusions still hold after the 1% and 2% tail-reduction treatments. In addition, the R^2^ of the robustness tests, except for individual models, also remain around 0.8, again proving the robustness of the above conclusions.

#### Endogeneity test

In addition, the non random selectivity of the cities where the policy is implemented and the possible reverse causality in the model will bring some endogenous problems and interfere with the regression results. In this study, the instrumental variable method is used to alleviate the above problems. The selection of instrumental variables should meet the two conditions of relevance and exclusivity, and have certain historical or natural attributes. As the predecessor of the PTFZs, the national comprehensive bonded zone is easier to be approved to set up a PFTZs, meeting the relevant conditions. Meanwhile, the special customs supervision area of the national comprehensive bonded zone was set up relatively early, which has relatively little impact on the carbon emissions of each city during the sample period of this study, meeting the exclusive and historical conditions. Therefore, whether a city has a national comprehensive bonded area is selected as a tool variable, and it is interacted with the variable Time_t_ in the above benchmark model to adapt to the panel data in this study. The results of the two stages are shown in Table 4. In the estimation results of the first stage, IV × Time_t_ is significantly positive correlated at the 1% statistical level, and the premise of correlation is met; The statistic of F test is 512.22, which is significantly greater than the critical value, indicating that there is no weak instrumental variable problem. In the second stage of regression results, the estimated coefficient of Policy_it_ is − 0.030, and it has passed the 5% significance test, which further indicates that the inhibition effect of the establishment of PFTZs on carbon emissions is still significant after reducing potential endogenous risks.

**Table 4:** Endogenetic test.

	I	II
*Policy*_*it*_		− 0.030**
		(− 2.25)
*IVTime*_*t*_	0.978***	
	(10.01)	
*Control*	Yes	Yes
*Cons*	0.044**	− 2.203***
	(2.13)	(− 4.75)
*N*	3976	3420
*F-value*	1298.35	
*Z-value*		512.22

### Heterogeneity test

On the basis of the above research, this study further examines the possible regional heterogeneity, urban scale heterogeneity, resource endowment heterogeneity and openness heterogeneity of institutional openness's inhibition on urban carbon emissions.

From the perspective of urban geographical endowment, the inhibition of PFTZs on urban carbon emissions has obvious regional heterogeneity. From the results of columns (1)–(3) in Table 5, the establishment of PFTZs in the eastern, central and western regions will reduce carbon emissions, but the inhibition effect of the eastern coastal areas is not significant. Only the estimated coefficients in the central and western regions pass the 1% significance test, and the emission reduction effect of the western PFTZs is the highest. This may be due to the fact that the eastern coastal areas, relying on their own labor, land and other factors' price advantages, have become the concentration of global co processing and export, forming an extensive growth mode inertia, and the emission reduction effect is not significant.

**Table 5:** Heterogeneity analysis.

	Regional heterogeneity			City size			Resource endowment		External dependence	
	Eastern	Central	Western	Large	Medium	Small	Resource	Non-Resource	High	Low
*Policy*_*it*_	− 0.009	− 0.107***	− 0.119***	− 0.013	− 0.042**	− 0.100***	− 0.118***	− 0.072***	− 0.054***	− 0.072***
	(− 0.70)	(− 4.57)	(− 4.97)	(− 0.29)	(− 2.02)	(− 4.64)	(− 4.86)	(− 4.23)	(− 3.09)	(− 4.23)
*lnfdi*	− 0.444**	− 0.005	− 0.104	− 0.303	− 0.190	− 0.929	0.038	− 0.252	− 0.103	− 0.252
	(− 2.34)	(− 0.05)	(− 0.93)	(− 1.26)	(− 0.67)	(− 0.89)	(0.30)	(− 0.22)	(− 1.14)	(− 0.22)
*lnpgdp*	0.867***	0.676*	− 0.307	1.451**	0.041	0.548	0.095	0.292	0.904***	0.292
	(3.89)	(1.76)	(− 0.87)	(2.37)	(0.13)	(1.55)	(0.25)	(0.91)	(2.99)	(0.91)
*lnpgdp*^2^	− 0.037***	− 0.030*	0.013	− 0.065**	0.002	− 0.026	− 0.003	− 0.014	− 0.040***	− 0.014
	(− 3.56)	(− 1.65)	(0.76)	(− 2.40)	(0.12)	(− 1.50)	(− 0.17)	(− 0.88)	(− 2.88)	(− 0.88)
*lnsec*	− 0.030	0.054	0.086*	0.219	0.010	0.088***	0.089**	0.079**	0.050	0.079**
	(− 0.53)	(1.41)	(1.66)	(1.33)	(0.16)	(2.65)	(2.16)	(2.28)	(0.86)	(2.28)
*lnfae*	0.019*	− 0.030*	0.031	0.011	0.024	− 0.004	0.003	0.025	− 0.005	0.025
	(1.67)	(− 1.71)	(1.33)	(0.83)	(1.37)	(− 0.31)	(0.19)	(1.63)	(− 0.39)	(1.63)
*lngov*	− 0.013	− 0.019	− 0.007	− 0.045	− 0.011	− 0.023	− 0.040*	− 0.029*	− 0.021*	− 0.029*
	(− 1.26)	(− 1.27)	(− 0.47)	(− 0.62)	(− 0.55)	(− 1.62)	(− 1.88)	(− 1.86)	(− 1.66)	(− 1.86)
*lnfina*	0.033**	0.011	0.014	0.099	0.017	0.027	0.063**	0.041*	0.023	0.041*
	(2.26)	(0.65)	(0.83)	(1.56)	(0.73)	(1.51)	(2.45)	(1.77)	(1.58)	(1.77)
*lnper*	− 0.004	− 0.055	0.062	0.060*	− 0.006	− 0.002	− 0.013	0.015	− 0.014	0.016
	(− 0.33)	(− 1.63)	(0.37)	(1.88)	(− 0.35)	(− 0.07)	(− 0.70)	(0.69)	(− 0.73)	(0.69)
*cons*	− 1.952*	− 0.766	3.688*	− 4.315	2.461	− 0.525	1.910	0.752	− 1.961	0.752
	(− 1.65)	(− 0.38)	(1.89)	(− 1.23)	(1.41)	(− 0.30)	(0.99)	(0.46)	(− 1.20)	(0.46)
*City FE*	Yes	Yes	Yes	Yes	Yes	Yes	Yes	Yes	Yes	Yes
*Year FE*	Yes	Yes	Yes	Yes	Yes	Yes	Yes	Yes	Yes	Yes
*R*^2^	0.887	0.792	0.861	0.813	0.845	0.815	0.814	0.823	0.817	0.823
*N*	1400	1400	1176	266	1372	2338	1596	1988	1988	1988

The industrial structure and resource allocation level of different cities are also different. Therefore, according to the New Classification List of Chinese Cities, this study evaluates the emission reduction effect of institutional openness on different city scales by taking the new first tier and first tier cities as large cities, the second tier and third tier cities as medium-sized cities, and the fourth and fifth tier cities as small cities. As shown in Table 5, the setting of PFTZs in large, medium and small cities has significantly reduced urban carbon emissions. Among them, small and medium-sized cities have significant policy effects, while the coefficient of Policy_it_ of large cities is not significant.

From the perspective of resource endowment heterogeneity, the establishment of PFTZs has a very significant role in reducing emissions in both resource-based cities and non resource-based cities, and the impact of resource-based cities is greater. The reason may be that the economic activities of resource-based cities are more dependent on resources and energy due to the influence of their own factor endowment. The setting of PFTZs injects new impetus into resource-based cities to make them have more reasonable resource allocation and mitigate the negative effects of resource mismatch, especially the environmental damage caused by excessive energy consumption.

The establishment and development of PFTZs will also be affected by the degree of dependence outside the city to a certain extent. Based on the median proportion of total foreign investment in GDP from 2006 to 2019, this study divides the sample into regions with high external dependence and regions with low external dependence, and evaluates the policy effect of PFTZs in the two types of regions. As shown in columns (8)–(9), the estimated coefficients of the two policy dummy variables are − 0.054 and − 0.072, and both are significant at the level of 1%. It is worth mentioning that PFTZs plays a better role in reducing emissions in regions with low external dependence. To sum up, H3 is valid.

### Further analysis

The close economic ties between cities in China make the convenience of the related regions in the macroeconomic operation show a strong correlation. And the external characteristics of environmental pollution itself also make CO_2_ emissions have a certain geo spatial correlation. The formal signing of the Regional Comprehensive Economic Partnership Agreement (RCEP) has further enhanced the complexity and economic relevance of China's FTA network. In view of this, it is necessary to explore the possible spatial dependence of the PFTZs when investigating its impact on carbon emissions. In addition, the economics of spatial geography also shows the relevance between things. Therefore, this study further explores the spatial effect of PFTZs in carbon emission reduction from a spatial perspective, and constructs the following spatial autocorrelation difference in differences model (SAR-DID):

5 $$ CO_{2it} = \alpha_{0} { + }\alpha_{1} Policy_{it} + CO_{2it} \times W + Policy_{it} \times W + \alpha \sum X_{it} + \mu_{i} + \eta_{t} + \varepsilon_{it} $$

*W* is the spatial weight matrix, and other variables have the same meaning as the above benchmark model. Before building the spatial measurement model, first set the spatial weight matrix. This study selects the adjacent space weight matrix (*W*_*1*_), geographical space weight matrix (*W*_*2*_), economic space weight matrix (*W*_*3*_) and economic geography nested space weight matrix (*W*_*4*_) to explore the spatial effect of the emission reduction effect of PFTZs under geographical and economic attributes respectively. Where, the element of *W*_*1*_ is assigned as 1 for the adjacent area, otherwise it is 0; The element of *W*_*2*_ is the reciprocal of the shortest expressway kilometers of the two cities; The element of *W*_*3*_ is the reciprocal absolute value of the difference between cities' per capita GDP. In addition, considering that there may be errors in measuring the spatial correlation between regions with a single geographical distance and economic correlation, this study further constructs a weighted economic geography nested spatial weight matrix *W*_*4*_ that considers both economic distance and geographical distance^15^, specifically: $W_{4} = \varpi W_{2} + 1{ - }\varpi W_{3}$, $\varpi$ is the weight, and the value is 0.5. It should be pointed out that the four spatial weight matrices mentioned above are all subject to variable standardization. The results are shown in Table 6. Under the four spatial weight matrices, the estimated coefficients of the policy dummy variable Policy_it_ are significantly negative and pass the 1% significance test. This shows that after the spatial factors are included in the model, the emission reduction effect of the PFTZs is still significant, and the emission reduction effect under the economic attribute is the strongest. The coefficient of W_ CO_2_ is significantly positive, indicating that the carbon emissions of each city significantly affect the carbon emissions of other cities through geographical and economic correlation, showing a spatial spillover effect of “prosperity for all”. H4 is verified.

**Table 6:** Spatial econometric model regression.

	W_1_	W_2_	W_3_	W_4_
*Policy*_*it*_	− 0.014***	− 0.034***	− 0.056***	− 0.053***
	(− 2.45)	(− 5.04)	(− 5.85)	(− 6.11)
*W_ CO*_*2*_	0.743***	3.363***	0.604***	0.863***
	(7.42)	(12.92)	(3.76)	(5.42)
*lnfdi*	− 0.038	− 0.046	− 0.303***	− 0.156***
	(− 1.06)	(− 1.11)	(− 5.03)	(− 2.83)
*lnpgdp*	0.442***	0.332***	0.432***	0.349***
	(1.03)	(6.54)	(5.90)	(5.26)
*lnpgdp*^2^	− 0.20	− 0.015***	− 0.015***	− 0.015***
	(− 9.67)	(− 6.26)	(− 4.32)	(− 4.57)
*lnsec*	0.047***	0.048***	0.021*	0.051***
	(5.37)	(4.66)	(1.68)	(4.51)
*lnfae*	0.003	− 0.005	0.032***	0.011**
	(0.92)	(− 1.36)	(6.42)	(2.51)
*lngov*	− 0.006	− 0.014***	0.021***	− 0.003
	(− 1.52)	(− 1.36)	(4.54)	(− 0.60)
*lnfina*	0.013***	0023***	0.006	0.016***
	(3.12)	(4.61)	(1.06)	(3.04)
*lnper*	− 0.009	− 0.018**	0.017	0.006
	(− 1.21)	(− 2.17)	(1.38)	(0.57)
*City FE*	Yes	Yes	Yes	Yes
*Year FE*	Yes	Yes	Yes	Yes
*R*^2^	0.571	0.100	0.755	0.754
*N*	3976	3976	3976	3976

### Impact mechanism analysis

The above empirical analysis results show that institutional openness can effectively curb urban carbon emissions. Based on the above mechanism analysis, this study further explores whether technological progress plays an intermediary role in the impact of institutional openness on urban carbon emissions. Among them, technological progress includes two perspectives: efficiency improvement oriented digital transformation and R&D innovation oriented green innovation capability. We build the following mediation effect model:

6 $$ CO_{2it} = \alpha_{0} { + }\alpha_{1} Policy_{it} + \alpha \sum {X_{it} } + \mu_{i} + \eta_{t} + \varepsilon_{it} $$

7 $$ M_{it} = \theta_{0} { + }\theta_{1} Policy_{it} + \theta \sum {X_{it} } + \mu_{i} + \eta_{t} + \varepsilon_{it} $$

8 $$ CO_{2it} = \zeta_{0} { + }\zeta_{1} Policy_{it} + \zeta_{2} M_{it} + \zeta \sum {X_{it} } + \mu_{i} + \eta_{t} + \varepsilon_{it} $$

$M_{it}$ is the intermediary variable of technological progress, including the ability of digital transformation and green innovation. Among them, the digital economy development index is taken as the proxy variable of digital transformation. Considering that R&D innovation depends more on education level, this study takes the interaction between green patent authorization and education level as the proxy variable of green innovation capability. The definitions of other variables are the same as above.

As shown in Table 7, from the perspective of technological progress represented by digital transformation, the coefficient of Policy_it_ in the two stages are 0.468 and − 0.143, both of which are significant at the 5% level. This shows that institutional openness can reduce urban CO_2_ emissions by promoting digital transformation, that is, the intermediary effect of digital transformation exists, and the proportion of intermediary effect in this path is 4.57%. From the perspective of technological progress represented by green innovation capability, the coefficients of Policy_it_ are 0.224 and − 0.162 respectively, and both pass the significance test with a statistical level of 5%, indicating that institutional openness can reduce urban CO_2_ emissions by increasing green innovation, that is, the intermediary effect of green technology innovation exists, and the intermediary effect under this path accounts for 6.65%. Combined with the regression results of the two, institutional openness can reduce urban CO_2_ emissions through technological progress, including digital transformation and green innovation capabilities. H4 holds.

**Table 7:** Mechanism analysis.

	Digital transformation		Green technology innovation	
	Digital economy index	carbon emission	Number of green patents granted	carbon emission
*Policy*_*it*_	0.468***	− 0.143**	0.224***	− 0.162**
	(3.23)	(− 2.26)	(2.58)	(− 2.44)
*lndi×edu*		− 0.015**		
		(1.96)		
*lngp*				0.045***
				(3.14)
*Control*	Yes	Yes	Yes	Yes
*City FE*	Yes	Yes	Yes	Yes
*Year FE*	Yes	Yes	Yes	Yes
*Total Effect*		− 0.150**		− 0.152**
		(− 2.37)		(− 2.29)
*Direct Effect*		− 0.143**		− 0.162**
		(− 2.26)		(− 2.44)
*Indirect Effect*		− 0.007*		0.010**
		(− 1.67)		(1.99)
*Proportion of intermediary effect*		4.57%		6.65%
*R*^2^	0.163	0.408	0.024	0.396
*N*	3976	3976	3976	3976

## Discussion

Institutional openness will not only take a leading position in international economic rule-making, but also achieve a win–win situation for economic development and environmental governance by gradually aligning with global economic development and environmental improvement. Institutional openness brings new policies, regulations and management modes. In terms of policy inclination, government departments' financial support for science and technology enables enterprises to reduce the risk of innovation to a certain extent, thus encouraging the generation of technological innovation. In addition, the introduction of preferential policies for the introduction of talents, such as the special support policy for the new area of Shanghai Lingang, attracts high-quality resources and technologies from the surrounding areas, generating a “siphon effect”, accelerating the development and application of new industries and green technologies^65^, and have an impact on the total scale of the city's economy and overall technological capability after maturity. This “scale effect” and “technology spillover effect” will have a reverse effect on the surrounding areas, comprehensively enhancing the overall green development level of the region^66^.

In terms of management mode, in July 2021, the “Hainan Free Trade Port Special Management Measures for Cross-border Trade in Services (Negative List) (2021 Edition)” was officially announced, which is the first negative list for cross-border trade in services in China, and is a major breakthrough in the management mode of China's trade in services, truly promoting mutual progress of openness and reform.

In recent years, academic research around energy conservation and emission reduction has continued to increase, which is significant for enriching theoretical and empirical studies in related fields. The literature has been well analyzed in terms of traditional factors such as industrial structure, energy structure and innovation capacity on CO_2_ emissions^67–69^, but it has not been explored in depth from the perspective of institutional openness, thus lacking a scientific response to carbon emission reduction in a macro context. Du et al. pointed out that in terms of the impact of foreign openness on We find empirical evidence is limited in the study of the mechanism of the impact of foreign openness on CO_2_ emissions^70^. In this paper, we construct a research framework of institutional openness and low carbon development to explore new mechanisms and new ways of carbon emission reduction in the context of institutional openness at both theoretical and empirical levels. The relationship between openness and environmental quality is a combination of multiple effects The exploration of the relationship between the two can help build a regulated trade market with sustainable development and green development norms, which in turn can contribute to national cleaner production in the globalization process^71^.

Relative to previous studies, the findings of this paper have both similarities and significant differences. In terms of similarity, trade openness suppresses urban CO_2_ emissions, which is consistent with Ma and Wang (2021)^72^. Providing institutional public goods to the outside world and achieving higher levels of trade openness are important ways to participate in global economic governance, and are key ways to improve cleaner production performance, mitigate climate change, and accelerate the low-carbon transition. This study finds that there is some heterogeneity and spatial correlation in the inhibitory effect of institutional openness on urban carbon emissions. Among them, the emission reduction effect is stronger in central and western regions, medium and small cities, resource-based cities, and cities with a low share of foreign direct investment. And the neighboring cities can influence each other. In other words, cities should consider not only the development characteristics of different cities themselves, but also the influence of surrounding cities when formulating system openness related trade policies, which was neglected by previous studies.

This paper has further findings in this paper in the mechanism analysis. The existing literature in the exploration of the impact paths of the environmental effects of opening up to the outside world lacks the integration of the contemporary context^73^. We argue that technological progress is an important pathway for institutional conduct of carbon reduction. Its adoption includes digital transformation oriented to efficiency improvement and green innovation capability oriented to R&D innovation. The new round of technological revolution has brought a great impact on the global economic governance order, especially under the influence of the current new crown pneumonia, and digital technology has gradually become an important driving force of the new round of globalization. Digital technology helps institutional openness, which not only reduces the cost of enterprises in foreign trade activities and improves production and operation efficiency. And it will also provide technical support for the green transformation of their own economies through technology interaction, technology absorption and technology transformation, and directly or indirectly reduce urban carbon emissions. Therefore, while gradually promoting system-based openness, we should pay more attention to improving the level and quality of openness and devote ourselves to driving the green development of the local economy, which is the key to achieving the dual goals of openness and low carbon.

Based on the existing studies, we believe that the research perspective, data measurement and empirical methods can be consolidated and expanded in the future, combined with the discussion of the emission reduction effect of institutional openness in this paper. In terms of research perspectives, there is no unified definition of the measurement standard of institutional openness, and this paper takes the setting of Pilot Free Trade Zones as the entry point for discussion, but there are still certain shortcomings. Future research can be extended on this basis to comprehensively and systematically construct the framework of institutional openness for emission reduction. In terms of data measurement, this paper uses particle swarm optimization-back propagation (PSO-BP) algorithm to calculate urban CO_2_ emissions based on remote sensing dataset of nighttime light images, which is scientific and accurate to a certain extent, but there is still a possibility of overestimation or underestimation due to the limitation of data availability. Therefore, a more scientific and reasonable carbon emission measurement system should be established, which can help policy makers better grasp the overall carbon emission situation of cities and use it to formulate corresponding emission reduction policies. Further, a more subdivided administrative unit is very helpful for the government to measure the regional emission reduction potential, which is one of the directions that can be further studied in the future. In terms of empirical methods, although different econometric methods are used to estimate the model and the robustness of the empirical results is effectively argued, the paper does not cover much about the possible nonlinearity problem. Moreover, the multi-period difference in difference (DID) method can only reduce the endogeneity problem to a certain extent, and the accuracy of the model evaluation may be affected. Therefore, future research can further quantitatively analyze the nonlinear effect relationship existing between the two with threshold regression models, breakpoint regression models, spatial threshold regression models, etc.

## Conclusion and policy implications

###  Research conclusion

Institutional openness is a major reform implemented by China in response to the global economic and trade development situation, and is also an important exploration of China in exploring the path of opening up to the outside world. Since 2013, China has successively set up 21 Pilot Free Trade Zones (PFTZs), gradually forming a new pattern of institutional openness in multiple fields, at multiple levels and in all directions. China's economy has entered a new stage of development, and economic development is gradually changing from a sloppy model of pursuing growth rate to an internal model of pursuing structural adjustment and environmental efficiency, and trade openness and energy conservation and emission reduction have become the two “main themes” of China's sustainable development. Based on this, this study is based on the impact of institutional openness on China's low carbon economic transformation. Based on 284 prefecture level cities in China from 2006 to 2019 as research samples, the establishment of PFTZs is taken as a quasi natural experiment, and the emission reduction effect of institutional openness is evaluated by building a gradual dual difference model.

The research finds that: first, institutional openness significantly suppresses urban CO_2_ emissions, and this finding holds after excluding possible non-random selection factors, other policy influences and endogeneity; Second, there is a certain heterogeneity in the inhibition effect of pilot policies of PFTZs on urban carbon emissions. Compared with the eastern region, large cities, non resource based cities and cities with a high proportion of foreign investors, the central and western regions, small and medium-sized cities, resource based cities and cities with a low proportion of foreign direct investment have a stronger role in reducing emissions; Thirdly, after considering the geographical and spatial factors, institutional openness still has significant emission reduction effect, and the effect under the economic attribute is the strongest; Fourth, the analysis of the mechanism of institutional openness affecting urban carbon emissions shows that the PFTZs achieves carbon emissions reduction through technological progress, including two paths: efficiency oriented digital transformation and R&D oriented green innovation capability improvement.

### Policy implications

The above conclusions can provide some important policy implications for institutional openness to promote the transformation of low-carbon economy:

First, build a dual wheel drive mechanism of institutional openness and low-carbon development to achieve the “perfect combination” of regional development strategies and energy conservation and emission reduction goals. We will continue to improve the quantity and quality of the development of PFTZs, provide better development opportunities for institutional openness, and provide a strong guarantee for international economic and trade partners to communicate in trade, investment and other fields. At the same time, we will stimulate the economic and trade vitality of our economic partners to the greatest extent, effectively improve the efficiency of resource allocation and energy utilization, accelerate the pace of China's integration with global economic development and green and low-carbon development, maximize benefits and minimize carbon emissions, and thus help build a beautiful China with “golden mountains and silver mountains” and “green waters and green mountains”^74^.

Second, build a high-quality Free Trade Zones network, establish a synergistic cooperation mechanism, and form a regional synergy for low-carbon development under system openness. Due to the existence of “learning effect”, “demonstration effect” and “economic correlation effect”, urban carbon emissions have a significant positive spatial spillover, so each region will be indirectly affected by geographical and economic correlation regions when implementing energy conservation and emission reduction policies. Especially in the context of institutional openness, the network members of the Free Trade Zones have closer exchanges, and it is imperative to establish a common low-carbon development. Among them, the government should fully recognize the key role of regional coordinated green development for individual cities to achieve carbon emission reduction, actively build emission reduction information sharing network, share low-carbon development experience, and strengthen the in-depth docking of regional green development to form a long-term effective cooperative operation mechanism, ultimately minimize the cost and maximize the benefits of overall and internal individual emission reduction, and achieve a “win–win” for economic development and environmental protection.

Third, we should seize the new opportunities brought by digital technology and fully release the digital dividend. Digital technologies with big data, cloud computing, 5G base stations, artificial intelligence and the Internet of Things as the core have injected new vitality into the high-quality development of the global economy. Members of the Free Trade Zones should rely on improving digital governance and regulatory rules, and focus on breaking through digital core technologies and training comprehensive digital technology talents to promote the development of digital international trade. With the help of digital technology, we will drive industrial green development and economic low-carbon transformation, accelerate the cultivation of new green and low-carbon drivers, and give full play to the carbon emission reduction effect of digital transformation in institutional openness.

Last, improve the green technology innovation ability, and effectively play the role of technological progress as the engine of carbon emission reduction^75^. Establish a sound green innovation technology system, stimulate the enthusiasm of market players for green R&D innovation through effective institutional arrangements and necessary incentives, and give full play to the positive externalities of green R&D innovation on the environment. Then government can also guide enterprises and residents to conduct green production and consumption behavior through policies, stimulate the research and development of green low-carbon products from both the demand side and the supply side, and stimulate the green innovation vitality of the market in an all-round and multi angle way.

## Supplementary Information

Supplementary Information 1.

Supplementary Information 2.

## Data Availability

All data generated or analysed during this study are included in this published article [and its supplementary information files].
