# Supplementary material for: Study on carbon emission reduction effect of institutional openness in China
Source: Sci Rep. 2023 Jan 5;13:254. doi: 10.1038/s41598-023-27442-5 (PMC9815685; doi:10.1038/s41598-023-27442-5)
Supplement: Supplementary file 1 — Supplementary Information 1. [file 41598_2023_27442_MOESM1_ESM.docx]

List of Responses

Dear Editors,

Thank you for your letter.

Figure 1, Figure 5, Figure 6 are created by the author, and the software was shown in the Figure 1 Figure 5, Figure 6, respectively.

We appreciate for Editors and Reviewers’ warm work earnestly, and hope that the correction will meet with approval.
